# Preconception thyroid function optimization and pregnancy outcomes in women with recurrent pregnancy loss: a real-world cohort study

**DOI:** 10.1530/EC-26-0215

**Published:** 2026-07-22

**Authors:** Heze Xu, Zhijiao Wang, Yijia Liu, Shanwei Xing, Hang Zhen, Jiapo Li, Chong Qiao

**Affiliations:** ^1^ Department of Obstetrics and Gynecology, Shengjing Hospital, China Medical University, Shenyang, Liaoning Province, China; ^2^ Key Laboratory of Maternal-Fetal Medicine of Liaoning Province, Shenyang, Liaoning, China; ^3^ Research Center of China Medical University Birth Cohort, Shenyang, Liaoning Province, China

**Keywords:** subclinical hypothyroidism, recurrent pregnancy loss, levothyroxine, pregnancy loss, miscarriage

## Abstract

**Background:**

The management of subclinical hypothyroidism with thyroid-stimulating hormone (TSH) levels between 2.5 and 4.0 mIU/L in women with recurrent pregnancy loss (RPL) remains controversial, as guidelines derived from general populations conflict. While a recent randomized trial confirmed the benefit of initiating levothyroxine (LT4) during early pregnancy, whether a more proactive strategy of preconception thyroid optimization improves outcomes in this high-risk group remains unknown.

**Methods:**

We conducted a retrospective cohort analysis of 896 women with RPL and elevated TSH (2.5–4.0 mIU/L) during pre-pregnancy, from the Recurrent Pregnancy Loss Cohort Study. Based on clinical records, participants were categorized into two groups: treated group (*n* = 636) and untreated group (*n* = 260). The primary outcome was miscarriage. Associations were assessed using Firth’s penalized-likelihood regression to address small-sample bias, with propensity score matching as a sensitivity analysis.

**Results:**

After adjustment for antiphospholipid syndrome, degree of education, gravidity, and parity, LT4 treatment was associated with a significantly lower risk of miscarriage (adjusted odds ratio (aOR) = 0.33; 95% CI: 0.20, 0.54; *P* < 0.001). A reduced risk was also observed for threatened abortion (aOR = 0.49; 95% CI: 0.35, 0.71; *P* < 0.001). Results were robust in the propensity score-matched analysis.

**Conclusion:**

In women with RPL and elevated TSH (2.5–4.0 mIU/L), a clinical care pathway involving preconception LT4 treatment was significantly associated with a reduced risk of miscarriage. This finding provides real-world evidence supporting the potential value of proactive preconception thyroid optimization in RPL, highlighting it as a priority strategy worthy of prospective investigation versus post-conception treatment initiation.

## Introduction

Subclinical hypothyroidism (SCH), characterized by elevated serum thyroid-stimulating hormone (TSH) with normal free thyroxine, is prevalent among women of reproductive age and associated with increased risks of adverse pregnancy outcomes (1, 2). A key clinical controversy surrounds the management of women with TSH levels in the gray zone between 2.5 and 4.0 mIU/L. The central question in preconception care for this group is whether such biochemical findings constitute a pathological condition warranting intervention or merely a benign laboratory variation. Reflecting evidence from the general population, major guidelines currently favor a conservative approach, not recommending routine levothyroxine (LT4) supplementation due to unproven benefit (3, 4).

However, directly extrapolating this non-intervention consensus from the general population to the unique high-risk group of women with recurrent pregnancy loss (RPL), defined as the loss of two or more consecutive pregnancies and affecting 1–2% of women (5), faces dual challenges. First, the assumption of biological homogeneity may not hold: RPL patients often present with active autoimmune backgrounds and an abnormal maternal–fetal interface microenvironment, making pregnancy maintenance potentially more sensitive to subtle thyroid hormone fluctuations (6, 7, 8). Second, and more clinically decisive, is the paradigm shift in risk–benefit assessment. For the general reproductive age population, tolerating a mildly elevated TSH to avoid overtreatment is reasonable. Yet, for RPL patients who have already experienced multiple pregnancy losses, the clinical value of any intervention that may reduce their absolute risk of another loss is dramatically amplified. Therefore, the applicability of this non-intervention consensus is questionable in RPL patients, a population with a high prevalence of SCH (9). Some early observational studies attempted to explore the value of treatment but failed to reach consistent conclusions due to small sample sizes or study design limitations (10, 11), leaving the management of SCH in RPL patients long guided by a lack of high-quality evidence.

This deadlock has recently been broken by a pivotal randomized controlled trial (RCT), which demonstrated that initiating LT4 treatment upon early pregnancy confirmation in RPL patients with SCH significantly improved live birth rates (12). This finding provided strong evidence for the efficacy of treatment, shifting the clinical question from whether to treat to how best to treat. Nevertheless, this landmark study naturally raises a more forward-looking question: if early treatment during pregnancy is effective, could further advancing the intervention to the preconception period, actively optimizing thyroid function to an ideal state (TSH < 2.5 mIU/L) before pregnancy, provide a more favorable environment for embryo implantation and early development, potentially leading to better outcomes than pregnancy-phase intervention? From a pathophysiological perspective, preconception intervention is theoretically more attractive because it aims to eliminate risk factors from the very beginning of pregnancy. However, unlike treatment during pregnancy, which is supported by RCT evidence, the effectiveness of this more proactive preconception optimization strategy remains entirely theoretical and lacks empirical evidence from large clinical cohorts.

Thus, this study aims to fill this critical evidence gap. Using real-world data from a large specialized RPL cohort, we systematically evaluated the association between the clinical pathway of ‘receiving preconception LT4 treatment and management to maintain TSH < 2.5 mIU/L’ and pregnancy outcomes. The purpose of this study was not to revalidate the efficacy of treatment but to prospectively explore the potential value of the more advanced management strategy of active preconception optimization. It aimed to offer RPL patients a potentially superior clinical decision option compared to the current standard (pregnancy-phase treatment) and to provide a solid basis for its future validation in prospective trials.

## Methods

### Study design and participants

This retrospective cohort study utilized data from the Recurrent Pregnancy Loss Cohort Study (RPLCS) between May 2015 and January 2026. The initial cohort comprised 3,787 patients with RPL, among whom 2,996 achieved pregnancy following etiological screening. This was a retrospective cohort study using data from a pre-established prospective cohort of women with RPL. The RPLCS collected detailed information on patients’ preconception clinical visits, etiological diagnoses for RPL, treatment regimens, post-conception examination results, various clinical outcomes, and pregnancy complications. We conducted an analysis on a subset of this cohort who met specific criteria for elevated TSH (2.5–4.0 mIU/L) before pregnancy. This cutoff was adopted in accordance with recommendations from the American Association of Clinical Endocrinologists, the American Thyroid Association, and the Endocrine Society to lower the upper reference limit for TSH during pregnancy (13, 14, 15). The exclusion criteria were as follows: i) history of thyroid surgery or radioiodine (I-131) therapy (*n* = 9); ii) hypothyroidism (defined as elevated TSH with decreased free thyroxine, *n* = 17) or TSH > 4 mIU/L (*n* = 51); total *n* = 68 (13); and iii) ectopic pregnancy (*n* = 3). Consequently, 896 pregnant women with a pre-pregnancy diagnosis of SCH were included in the final analysis.

### Exposure

The exposure was defined as the use of LT4 therapy before pregnancy. Women with SCH were divided into two groups: i) the treated group, those who received LT4 to maintain TSH < 2.5 mIU/L (*n* = 636), and ii) the untreated group, those who did not receive LT4 treatment prior to pregnancy (*n* = 260). In real-world clinical practice, women remained untreated for three main reasons: i) a clinical decision by the physician not to prescribe LT4 (*n* = 161), ii) patient’s refusal of treatment after being informed (*n* = 30), and iii) unplanned pregnancy occurring before the preconception evaluation was completed (*n* = 69). We acknowledge that these populations may differ significantly in terms of health-seeking behaviors, pregnancy intentionality, and adherence to medical advice. Although all patients in both groups underwent systematic preconception evaluation in a specialized RPL clinic, the treatment decision itself was not randomly assigned but was influenced by physician judgment, patient preference, and contingent events. Consequently, any association observed in this study is likely a mixture of the biological effect of LT4, patient characteristics related to the treatment decision, and unmeasured clinical nuances. Throughout our analyses, we deliberately use the term exposure rather than intervention to emphasize the observational nature of this study.

### Outcomes

Pregnancy was defined as a positive urine or serum human chorionic gonadotropin test or ultrasound-confirmed gestational sac. Ectopic pregnancy, hydatidiform mole, and failed embryo implantation were excluded.

The primary outcome was miscarriage, defined as spontaneous pregnancy loss before 20 weeks of gestation or fetal weight less than 500 g. Secondary outcomes included threatened miscarriage, gestational diabetes mellitus (GDM), and cervical cerclage.

Threatened miscarriage was assessed using two indicators: i) vaginal bleeding or brownish discharge that occurs before 13 weeks of gestation, excluding implantation bleeding and cervical lesions, and ii) sonographic fluid collection, which shows intrauterine fluid collection or an anechoic area around the gestational sac detected by ultrasound before 20 weeks.

Cervical cerclage performed during pregnancy due to cervical insufficiency was recorded as a secondary outcome. GDM was screened using an oral glucose tolerance test at 24 weeks of gestation. For outcomes diagnosed later in pregnancy (GDM or cervical cerclage), the denominator excludes women who experienced miscarriage prior to the gestational age window for that outcome.

### Covariates

Baseline information was collected, including maternal age, height, weight, spouse’s age, monthly household income, educational level, gravidity, and parity. These data were obtained during the patient’s first clinic visit by an outpatient assistant using a structured interview questionnaire. Details regarding gravidity and parity were further verified by the attending physician during consultation.

Patients with recurrent miscarriage underwent etiological screening during their visit. Given the limited sample size, this study focused on the following major etiologies: chromosomal abnormalities, endocrine disorders (thyroid dysfunction, polycystic ovary syndrome (PCOS), and diabetes), antiphospholipid syndrome (APS), and uterine anatomical abnormalities (16).

Chromosomal analysis was performed on both partners; abnormality in either partner was recorded as a chromosomal abnormality. Complete karyotyping was available for 86.4% of patients (*n* = 777). For 119 patients, only one partner underwent testing or no testing was performed, and these were recorded as missing values.

Thyroid function was assessed by measuring T3, FT4, and TSH, with specific assay methods as previously described in publications from this cohort (16, 17). PCOS was diagnosed according to the Rotterdam criteria (18). Diabetes was diagnosed based on two separate fasting plasma glucose measurements ≥7.0 mmol/L.

APS was evaluated based on anticardiolipin (aCL) antibodies and anti-β2-glycoprotein I antibodies (aβ2-GPI). All patients with an initial positive result were asked to undergo repeat testing at least 12 weeks apart. APS was diagnosed when persistently high antibody titers were confirmed on two occasions.

Congenital uterine anomalies (septate, bicornuate, unicornuate uterus) were identified via ultrasound. Acquired uterine abnormalities, such as intrauterine adhesions, were initially screened by ultrasound and confirmed by hysteroscopy.

In selecting adjustment variables, we considered thyroid peroxidase antibody (TPOAb) and thyroglobulin antibody (TgAb) status. While TPOAb or TgAb positivity is a known risk factor for miscarriage, it also serves as a key determinant in the clinician’s decision to initiate levothyroxine treatment. To preserve this real-world clinical decision logic and avoid introducing bias by adjusting for an intermediate variable lying on the causal pathway between treatment decision and outcome, we chose not to include thyroid autoimmune status in the final multivariable adjustment model. Our core model aimed to estimate the overall association of the entire clinical decision pathway, including decisions informed by the status of TPOAb and TgAb.

### Statistical analysis

Continuous variables are presented as mean ± standard deviation if normally distributed, or as median (interquartile range) otherwise. Normality was assessed using the Shapiro–Wilk test. Categorical variables are presented as number (percentage). Differences in baseline characteristics between the treated and untreated groups were compared using the independent Student’s *t* test (or Mann–Whitney U test for non-normally distributed data) for continuous variables and the chi-square test (or Fisher’s exact test for expected cell counts <5) for categorical variables.

Given the limited number of events for the primary outcome, which violates the conventional rule of thumb for multivariable logistic regression, we employed Firth’s penalized-likelihood logistic regression to reduce small-sample bias and obtain more reliable estimates (19). This method provides finite and less biased odds ratios (ORs) in the presence of rare events or complete separation. The model was adjusted for the following pre-specified potential confounders identified as imbalanced at baseline (*P* < 0.10): APS status, education level, PCOS, gravidity, and parity. Results are reported as ORs with 95% confidence intervals (CIs).

To strengthen causal inference, a propensity score matching (PSM) sensitivity analysis was conducted. Propensity scores were estimated from potential confounders, identified as imbalanced at baseline (*P* < 0.10) or deemed clinically important, and 1:1 optimal matching was applied. Balance was verified (all standardized mean differences (SMDs) < 0.1), and outcomes were analyzed in the matched cohort using conditional logistic regression. Secondary outcomes were analyzed similarly. The PSM cohort was constructed to assess the effect of treatment on all pregnancy outcomes from the time of cohort entry. For outcomes that are necessarily diagnosed later in gestation (GDM and cervical cerclage), the analysis was conditioned on surviving without miscarriage until the gestational age at which these outcomes are typically ascertained. This approach preserves the baseline comparability of the matched cohort while acknowledging the appropriate at-risk population for each outcome.

In summary, our analytical strategy was twofold: first, to use a bias-reduced model (Firth’s regression) for unbiased estimation in the full cohort; second, to emulate a randomized trial via PSM as a robust sensitivity check.

All statistical tests were two-sided, and a *P* value of <0.05 was considered statistically significant. All analyses were performed using R software (version 4.5.1), using the ‘logistf’ package for Firth’s regression and the ‘MatchIt’ package for PSM.

## Results

### Study population and baseline characteristics

Of the initial 3,787 women in the RPLCS cohort, 896 pregnant women with elevated TSH were eligible for this analysis. Among them, 636 women (71.0%) received the standard treatment (treated group), while 260 women (29.0%) did not receive treatment (untreated group). The detailed flow of participant selection is depicted in Supplementary Fig. 1 (see section on Supplementary materials given at the end of the article).

Baseline demographics are summarized in Table 1. The two groups were largely comparable; however, significant differences were noted in degree of education (*P* < 0.001), gravidity (*P* < 0.001), and parity (*P* < 0.001).

**Table 1:** Baseline characteristics of pregnant women with elevated TSH, stratified by treatment adherence.

Characteristic	Total cohort	Treated group	Untreated group	*P* value
	(*n* = 896)	(*n* = 636)	(*n* = 260)	
Maternal demographics				
Age (years), mean ± SD	31.6 (3.6)	31.6 (3.4)	31.6 (4.0)	0.99
Spouse’s age (years), mean ± SD	32.1 (4.2)	32.1 (4.2)	32.2 (4.2)	0.95
BMI (kg/m^2^), mean ± SD	22.6 (3.2)	22.6 (2.9)	22.4 (3.7)	0.84
Monthly income, *n* (%)				
– Low (<¥7,000)	151 (16.9)	109 (17.1)	42 (16.2)	0.62
– Medium (¥7,000-¥10,000)	655 (73.1)	467 (73.4)	188 (72.3)	
– High (>¥10,000)	90 (10.0)	60 (9.4)	30 (11.5)	
Education level, *n* (%)				
– High school or below	244 (27.2)	157 (24.7)	87 (33.5)	<0.001
– Bachelor’s degree	566 (63.2)	445 (70.0)	121 (46.5)	
– Postgraduate or above	86 (9.6)	34 (5.3)	52 (20.0)	
Obstetric history				
Gravidity, *n* (%)				
– 2	542 (60.5)	406 (63.4)	136 (52.3)	<0.001
– 3	226 (25.2)	136 (21.4)	90 (34.6)	
– ≥4	128 (14.3)	94 (14.8)	34 (13.1)	
Parity, *n* (%)				
– 0 (Nulliparous)	848 (94.6)	594 (93.4)	254 (97.7)	0.003
– ≥1	48 (5.4)	42 (6.6)	6 (2.3)	
Days of preconception treatment, median (IQR)	327 (333)	329 (310)	319 (363)	0.52

BMI, body mass index; SD, standard deviation; IQR, interquartile range. *P* values for continuous variables were derived from the independent *t*-test (normally distributed) or Mann–Whitney U test (non-normally distributed). *P* values for categorical variables were derived from the chi-square test or Fisher’s exact test, as appropriate.

Etiological characteristics are shown in Table 2. The prevalence of APS was lower in the treated group compared to the untreated group (9.9 vs 18.5%, *P* = 0.006), while polycystic ovary syndrome was also less frequent in the treated group (6.3 vs 10.4%, *P* = 0.048). Conversely, thyroid autoimmunity, indicated by TPOAb and TgAb positivity, was significantly higher in the treated group (both *P* < 0.001). No significant differences were found for other factors, such as parental abnormalities, diabetes mellitus, or uterine anatomical abnormalities (all *P* > 0.05).

**Table 2:** Etiological screening profile of the recurrent pregnancy loss cohort, stratified by levothyroxine treatment.

Etiological factor	Total cohort	Treated group	Untreated group	*P* value
	(*n* = 896)	(*n* = 636)	(*n* = 260)	
Any parental abnormality, *n*/N (%)	104/777 (13.4)	74/561 (13.2)	30/216 (13.9)	0.89
Antiphospholipid syndrome	111 (12.4)	63 (9.9)	48 (18.5)	0.006
Endocrine dysfunction				
– Polycystic ovary syndrome, *n* (%)	67 (7.5)	40 (6.3)	27 (10.4)	0.048
– Diabetes mellitus, *n* (%)	14 (1.6)	11 (1.7)	3 (1.2)	0.57
Uterine anatomical abnormalities				
– Congenital (e.g. septate uterus)	82 (9.2)	56 (8.8)	26 (10.0)	0.66
– Acquired (e.g. intrauterine adhesions)	183 (20.4)	132 (20.8)	51 (19.6)	0.77
Thyroid autoimmune				
– TPOAb positive	104 (11.6)	90 (14.2)	14 (5.4)	<0.001
– TgAb positive	102 (11.4)	92 (14.5)	10 (3.8)	<0.001

Data are presented as *n* (%). *P* values were derived from the chi-square test or Fisher’s exact test, as appropriate, for comparisons between the treated and untreated groups. *N* denotes the number of patients with available data for each specific test. Data on parental karyotyping were unavailable for 45 patients (29 in the treated group and 16 in the untreated group). TPOAb, thyroid peroxidase antibody; TgAb, thyroglobulin antibody.

### Primary outcome: miscarriage

In the primary analysis using Firth’s regression adjusted for education level, antiphospholipid syndrome status, polycystic ovary syndrome, gravidity, and parity, adherence to treatment was significantly associated with a reduced risk of miscarriage (Table 3). The incidence of miscarriage was 5.8% (37/636) in the treatment group, compared with 16.5% (43/260) in the untreated group. The adjusted odds ratio (aOR) was 0.33 (95% CI: 0.20, 0.54; *P* < 0.001). Miscarriage details are summarized in Table 4. The treated group had a significantly lower rate of biochemical pregnancy compared with the untreated group (1.7 vs 4.6%; RR: 0.37; 95% CI: 0.17, 0.84; *P* = 0.022). Regarding miscarriage, the treated group demonstrated a significantly reduced risk of fetal demise or spontaneous abortion (4.1 vs 11.9%; RR: 0.34; 95% CI: 0.21–0.57; *P* < 0.001). In exploratory analyses stratified by chromosomal testing status, the treated group had lower rates of miscarriage with normal chromosomes (*P* = 0.044) and miscarriage with abnormal chromosomes, although the latter did not reach statistical significance (*P* = 0.058) (Table 4).

**Table 3:** Associations between levothyroxine treatment and pregnancy outcomes.

Outcome	Treatment group	Untreated group	Crude OR	*P* value	Adjusted OR	*P* value
	(*n* = 636)	(*n* = 260)	(95% CI)		(95% CI)*	
Primary outcome						
Miscarriage, *n* (%)	37 (5.8%)	43 (16.5%)	0.31 (0.20, 0.50)	<0.001	0.33 (0.20, 0.54)	<0.001
Secondary outcomes						
Threatened abortion (vaginal bleeding or brownish discharge), *n* (%)	104 (16.4%)	75 (28.8%)	0.48 (0.34, 0.68)	<0.001	0.49 (0.35, 0.71)	<0.001
Perigestational/intrauterine fluid collection, *n* (%)	125 (19.7%)	126 (48.5%)	0.26 (0.19, 0.36)	<0.001	0.27 (0.20, 0.38)	<0.001
GDM, *n*/N (%)	83/597 (13.9%)	21/217 (9.7%)	1.50 (0.91, 2.49)	0.116	1.61 (0.97, 2.76)	0.067
Cervical cerclage, *n*/N (%)	30/597 (5.0%)	12/217 (5.5%)	0.90 (0.45, 1.80)	0.774	0.77 (0.38, 1.61)	0.467

OR, odds ratio; CI, confidence interval; GDM, gestational diabetes mellitus.

* Adjusted OR: derived from Firth’s penalized-likelihood logistic regression model, adjusted for education level, antiphospholipid syndrome status, polycystic ovary syndrome, gravidity, and parity.

All tests were two-sided. For GDM and cervical cerclage, the denominators (*n* = 597 in the treated group; *n* = 217 in the untreated group) exclude women who experienced miscarriage prior to the gestational weeks when these outcomes are typically assessed.

**Table 4:** Miscarriage details in the treated and untreated groups.

Outcome	Treated group	Untreated group	Risk ratio	*P* value
	(*n* = 636)	(*n* = 260)	(95% CI)	
Biochemical pregnancy, *n* (%)	11 (1.7%)	12 (4.6%)	0.37 (0.17–0.84)	0.022
Fetal demise or spontaneous abortion, *n* (%)	26 (4.1%)	31 (11.9%)	0.34 (0.21–0.57)	<0.001
Stratified by chromosomal testing				
- Without testing	7 (1.1%)	12 (4.6%)	0.24 (0.10–0.60)	0.002
- With normal chromosomes	5 (0.8%)	7 (2.7%)	0.29 (0.09–0.91)	0.044
- With abnormal chromosomes	14 (2.2%)	12 (4.6%)	0.48 (0.22–1.02)	0.058

Data are presented as *n* (%). CI, confidence interval. Miscarriages include all cases of miscarriage regardless of chromosomal testing status. The stratified results by testing status are exploratory and should be interpreted with caution due to potential selection bias in the decision to perform testing.

### Secondary pregnancy outcomes

Associations between treatment adherence and secondary outcomes are presented in Table 3. After adjustment, treatment was significantly associated with a lower risk of vaginal bleeding or brownish discharge (aOR = 0.49; 95% CI: 0.35, 0.71; *P* < 0.001) and subchorionic hematoma or intrauterine collection (aOR = 0.27; 95% CI: 0.20, 0.38; *P* < 0.001). No statistically significant association was observed for the risks of GDM (aOR = 1.61; 95% CI: 0.97, 2.76; *P* = 0.067) or cervical cerclage (aOR = 0.77; 95% CI: 0.38, 1.61; *P* = 0.467).

### Sensitivity analysis using propensity score matching

The results of the PSM sensitivity analysis corroborated the primary findings. Optimal matching successfully balanced all vital clinical covariates (all SMDs < 0.1), while a slight residual imbalance remained in the propensity score itself (SMD = 0.15). This resulted in 260 well-matched pairs (520 individuals).

As shown in Table 5, the treated group demonstrated a significantly lower rate of the primary outcome, miscarriage, compared with the untreated group (2.8 vs 15.1%; aOR = 0.44; 95% CI: 0.26, 0.77; *P* = 0.004). For secondary outcomes, the incidence of threatened abortion was markedly reduced in the treated group (17.0 vs 29.2%; aOR = 0.32; 95% CI: 0.20, 0.51; *P* < 0.001). Similarly, the treated group had a significantly lower occurrence of perigestational or intrauterine fluid collection (24.5 vs 49.1%; aOR = 0.31; 95% CI: 0.21, 0.46; *P* < 0.001).

**Table 5:** Associations between levothyroxine treatment and pregnancy outcomes in the propensity score-matched cohort.

Outcome	Treated group	Untreated group	Matched aOR	*P* value
	(*n* = 260)	(*n* = 260)	(95% CI)	
Primary outcome				
Miscarriage	3 (2.8)	16 (15.1)	0.44 (0.26, 0.77)	0.004
Secondary outcomes				
Threatened abortion (vaginal bleeding or brownish discharge)	18 (17.0)	31 (29.2)	0.32 (0.20, 0.51)	<0.001
Perigestational/intrauterine fluid collection	26 (24.5)	52 (49.1)	0.31 (0.21, 0.46)	<0.001

aOR, adjusted odds ratio; CI, confidence interval.

## Discussion

This study investigated the association between preconception LT4 treatment for elevated TSH and pregnancy outcomes in a cohort of women with RPL. The principal finding is that, after adjusting for key confounders, preconception LT4 treatment was associated with a substantially lower risk of miscarriage (aOR ≈ 0.33). This provides a clear, positive preliminary answer to the core question posed in our introduction regarding the value of actively optimizing preconception thyroid function in this high-risk population.

Our findings engage in a direct and important dialogue with the prevailing ‘no treatment’ consensus derived from general or infertile populations. The fundamental rationale of major guidelines against routine intervention for women with TSH levels between 2.5 and 4.0 mIU/L is that the absolute benefit in the general population is small and may not justify the risks of overtreatment (20, 21). However, our study suggests this rationale may not hold for the high-risk RPL subgroup. The 16.5% miscarriage rate in the untreated group of our cohort underscores the exceptionally high baseline risk in this population. In this context, an association indicating an ∼67% reduction in relative risk (corresponding to an aOR of 0.33) translates into an absolute risk reduction. Therefore, our data essentially pose an evidence-based challenge from a high-risk population to the one-size-fits-all consensus: when the baseline risk is sufficiently high, a modest relative benefit can transform into a clinically significant absolute benefit, potentially tipping the risk–benefit balance entirely and rendering intervention reasonable, if not necessary.

As an observational study, the nature of this association must be interpreted with caution. The core interpretation is not necessarily that LT4 medication per se caused the risk reduction but rather that a successful clinical pathway of preconception thyroid optimization identifies and contributes to a clinical state more conducive to pregnancy maintenance. This encompasses at least three possibilities: the first is direct physiological optimization of thyroid hormone levels at the maternal–fetal interface by LT4 (22, 23); second, the treatment act itself may select for patients with better health adherence and more thorough preconception preparation (healthy user effect) (24); and third, the physician’s decision to treat reflects a complex clinical judgment, constituting a key source of confounding by indication (25). For instance, our data showed a relatively lower TPOAb positivity rate in the untreated group, suggesting that clinicians might be more inclined to initiate treatment for SCH patients who are TPOAb-positive, as the autoimmune context strengthens the rationale for intervention (26, 27). Conversely, the higher prevalence of concomitant APS in the untreated group may reveal another clinical reality: when a clear, prioritized cause, such as APS, is present, the attention of both clinicians and patients may be diverted, potentially leading to the neglect or lower prioritization of mild SCH – a manifestation of the halo effect in clinical decision-making. Although we statistically controlled for known confounders, such as APS status, the subtler clinical reasoning driving treatment decisions cannot be fully measured. Nevertheless, even accounting for this confounding effect, the strength of the association signal strongly supports the critical role of thyroid function optimization in maintaining high-risk pregnancies.

Previous studies on SCH in RPL patients have yielded inconsistent conclusions. For example, the study by Hiroyuki Yoshihara *et al.* found no significant improvement in live birth rate with treatment, with an exceptionally wide CI for the adjusted risk estimate, suggesting potential statistical model instability due to a limited number of outcome events (11). Another study by Geneviève Leduc-Robert *et al.*, which compared a treatment group to a control group comprising a mixture of untreated SCH patients and euthyroid individuals, may have diluted the true treatment effect due to its analytical framework (10). These negative or inconclusive results highlight the extreme importance, in non-randomized designs, of precisely defining exposure and control groups and employing appropriate methods to control bias. In comparison, the strengths of our study are as follows: first, a pure population comparison, strictly limited to SCH patients with preconception TSH > 2.5 mIU/L, directly comparing treated versus untreated clinical pathways, avoiding confounding from population heterogeneity; second, a tailored methodological approach, applying Firth’s penalized-likelihood regression to address the small sample and rare event, yielding a more stable and less biased effect estimate than conventional logistic regression; third, a robust analytical strategy, with sensitivity analysis using PSM further supporting the reliability of the main finding. Therefore, the positive association evidence provided by our study constitutes an important methodological complement and deepening of the previously uncertain observational evidence.

However, the evidence landscape in this field was truly reshaped by a recent large RCT. This trial definitively demonstrated that initiating LT4 treatment in early pregnancy for RPL patients with SCH significantly increased live birth rates (12). This high-quality evidence unequivocally established treatment efficacy and advanced the core clinical question from ‘whether to treat’ to ‘how best to treat’. It is precisely on this foundation that the unique value of our study becomes clear: we did not seek to revalidate whether treatment is effective, the RCT has answered that affirmatively, but instead explored a more forward-looking clinical-scientific question: ‘could advancing the intervention timing from pregnancy to the preconception period, for active thyroid optimization, offer potential benefits beyond the established protocol?’ From a pathophysiological perspective, preconception intervention aims to create a reliable thyroid hormone environment for embryo implantation and very early development (23), theoretically offering an advantage over post-conception intervention. Our study systematically evaluated this active preconception optimization strategy using large-scale real-world data and found a strong correlation with pregnancy outcomes. Thus, our work forms a critical connecting link in the evidence chain: the RCT resolved the question of ‘efficacy of treatment during pregnancy’, while our work provides the highest-grade real-world data support to date for the hypothesis that ‘preconception intervention might be superior’.

The clinical implications of this study are twofold. First, for clinicians managing RPL, these data strongly suggest that when encountering an RPL patient with preconception TSH > 2.5 mIU/L, the mindset should shift from viewing this as an ‘ordinary finding’ to recognizing it as a ‘high-risk context’. Patients should be fully engaged in shared decision-making, discussing the limitations of current guidelines, the potential benefits and uncertainties suggested by our study, and whether to initiate LT4 treatment. Second, and more fundamentally, this study reveals an urgent clinical research need. Observational data, however compelling, cannot definitively establish causality. Therefore, we call for and urge the scientific community to prioritize a multicenter RCT specifically targeting RPL patients with preconception TSH levels between 2.5 and 4.0 mIU/L to conclusively determine the net benefit of preconception LT4 treatment in this specific population. The full value of our study lies in providing the solid preliminary evidence and clinical rationale necessary to justify such an essential RCT.

The conclusions of this study must be interpreted within the context of its inherent limitations. First, the retrospective observational design precludes definitive causal inference between treatment and outcome. Second, the non-randomized treatment assignment introduces confounding bias that is difficult to measure completely. Although we adjusted for measured baseline differences (e.g. APS) via statistical modeling, residual confounding from unmeasured factors driving physician or patient choice (e.g. varying levels of attention paid to mild biochemical abnormalities, unrecorded lifestyle factors) likely remains. Third, the internal heterogeneity of the untreated group is a specific weakness. This group combined various scenarios, including patient refusal of treatment and unplanned pregnancies. These subgroups may differ systematically in health consciousness, pregnancy planning, and baseline risk, and their pooled analysis might affect the purity of the effect estimate. In addition, our study lacks follow-up data on offspring. This limits our ability to assess potential long-term neurodevelopmental or other health outcomes in children born to mothers who received preconception LT4. Finally, although we employed Firth regression to mitigate small-sample bias, the absolute number of primary outcome events remains relatively low, which may limit the capacity for deeper subgroup analyses and affect the precision of the estimates.

In summary, in this large real-world study of RPL patients with SCH (TSH > 2.5 mIU/L), we found that the clinical pathway of selecting and completing preconception LT4 treatment and management to achieve TSH <2.5 mIU/L was associated with a significantly reduced risk of early pregnancy loss. While the aforementioned limitations necessitate caution in causal interpretation, this strong association signal, combined with the recent RCT evidence confirming the efficacy of pregnancy-phase treatment, jointly underscores the critical importance of thyroid function management in RPL clinical practice. Our findings challenge the current practice of simplistically applying treatment thresholds from the general population to this high-risk group and provide, for the first time, large-scale data support for the more proactive and prospective management strategy of ‘active preconception thyroid optimization’. This suggests that preconception counseling and management for RPL patients should place high importance on screening for and intervening in thyroid status. This study provides compelling preliminary evidence and clinical rationale for initiating such necessary research.

## Supplementary materials



## Declaration of interest

The authors declare that there is no conflict of interest that could be perceived as prejudicing the impartiality of the work reported.

## Funding

This work was supported by National Key R&D Program of China (2023YFC2705900), the National Natural Science Foundation of China (81370735, 81771610), the Science and Technology Project of Shenyang (20–205-4–004), the Livelihood Science and Technology Joint Project of Liaoning Province (2021JH2/10300123) and the Department of Education Basic Scientific Research Projects (LJ212410159092).

## Author contribution statement

HX and CQ conceived the study and administered the project. HX, ZW, YL, JL, and HZ curated the data. HX formulated the methodology. SX performed visualization. HX and JL wrote the manuscript. CQ acquired funding and supervised the study.

## Data availability

The data and code of this study are available from the corresponding authors on reasonable request.

## Ethics statement

This study was reviewed and approved by the Ethics Committee of China Medical University (2018PS381K). This study adhered strictly to the ethical guidelines outlined in the Declaration of Helsinki. All participants provided written informed consent for the use of their clinical data.

## Declaration of generative AI

This study did not utilize AI tools at any stage of the research process, including data collection, analysis, interpretation, or manuscript preparation. All methodologies and analyses were conducted by human researchers.
