# Supplementary material for: Preconception thyroid function optimization and pregnancy outcomes in women with recurrent pregnancy loss: a real-world cohort study
Source: Endocr Connect. 2026 Jul 22;15(7):e260215. doi: 10.1530/EC-26-0215 (PMC13393303; doi:10.1530/EC-26-0215)
Supplement: Supplementary file 1 [file EC-26-0215_supplementary_figure_1.pdf]

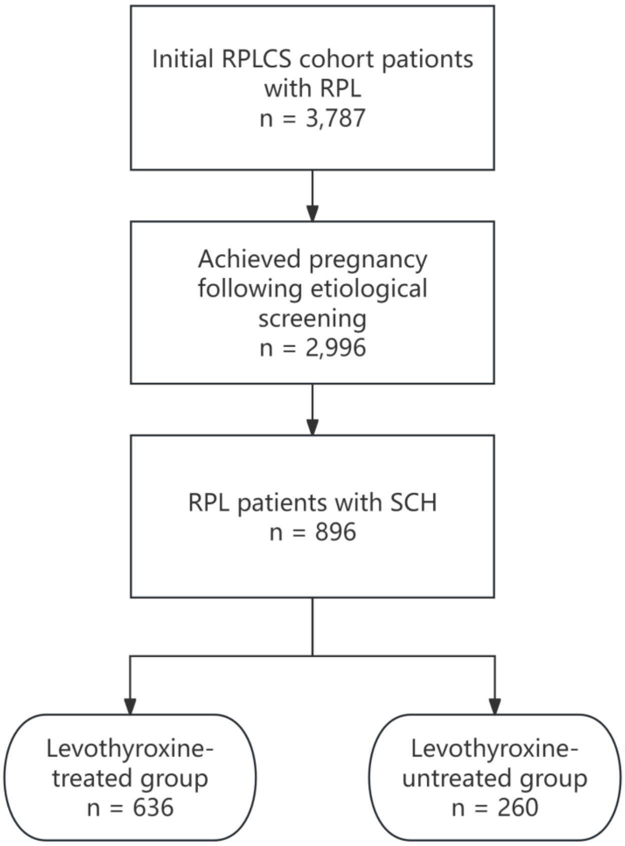

Supplementary Figure 1. Flowchart of Study Participant Selection.

Abbreviations: RPL, recurrent pregnancy loss; RPLCS, Recurrent Pregnancy Loss Cohort Study; SCH, subclinical hypothyroidism; TSH, thyroid-stimulating hormone.

*Clinical hypothyroidism was defined as elevated serum TSH accompanied by decreased free thyroxine.
